# The Phenazine 2-Hydroxy-Phenazine-1-Carboxylic Acid Promotes Extracellular DNA Release and Has Broad Transcriptomic Consequences in *Pseudomonas chlororaphis* 30–84

**DOI:** 10.1371/journal.pone.0148003

**Published:** 2016-01-26

**Authors:** Dongping Wang, Jun Myoung Yu, Robert J. Dorosky, Leland S. Pierson, Elizabeth A. Pierson

**Affiliations:** 1 Earth and Environmental Sciences, Los Alamos National Laboratory, Los Alamos, NM, 87544, United States of America; 2 Department of Plant Pathology and Microbiology, Texas A&M University, College Station, TX, 77843–2132, United States of America; 3 Department of Horticultural Sciences, Texas A&M University, College Station, TX, 77843–2133, United States of America; Robert Koch-Institute, GERMANY

## Abstract

Enhanced production of 2-hydroxy-phenazine-1-carboxylic acid (2-OH-PCA) by the biological control strain *Pseudomonas chlororaphis* 30–84 derivative 30-84O* was shown previously to promote cell adhesion and alter the three-dimensional structure of surface-attached biofilms compared to the wild type. The current study demonstrates that production of 2-OH-PCA promotes the release of extracellular DNA, which is correlated with the production of structured biofilm matrix. Moreover, the essential role of the extracellular DNA in maintaining the mass and structure of the 30–84 biofilm matrix is demonstrated. To better understand the role of different phenazines in biofilm matrix production and gene expression, transcriptomic analyses were conducted comparing gene expression patterns of populations of wild type, 30-84O* and a derivative of 30–84 producing only PCA (30-84PCA) to a phenazine defective mutant (30-84ZN) when grown in static cultures. RNA-Seq analyses identified a group of 802 genes that were differentially expressed by the phenazine producing derivatives compared to 30-84ZN, including 240 genes shared by the two 2-OH-PCA producing derivatives, the wild type and 30-84O*. A gene cluster encoding a bacteriophage-derived pyocin and its lysis cassette was upregulated in 2-OH-PCA producing derivatives. A holin encoded in this gene cluster was found to contribute to the release of eDNA in 30–84 biofilm matrices, demonstrating that the influence of 2-OH-PCA on eDNA production is due in part to cell autolysis as a result of pyocin production and release. The results expand the current understanding of the functions different phenazines play in the survival of bacteria in biofilm-forming communities.

## Introduction

Pseudomonads are well known for the production of a diversity of secondary metabolites, including phenazines that are essential for the control of plant diseases [1]. Phenazines are of particular interest because of their broad-spectrum antibiotic activity against diverse organisms from bacteria to eukaryotes, but also because they serve numerous functions that affect bacterial physiology and interactions with other organisms [2,3]. Phenazines comprise a large group of nitrogen-containing heterocyclic compounds that are synthesized only by bacteria, primarily *Pseudomonas* and *Streptomyces* species. Phenazines differ in their chemical and physical properties based on the type and position of functional groups present on the conserved three-ring structure [2]. Bacterial strains within the same species frequently differ in the types of phenazines they produce and often produce more than one phenazine derivative. Ultimately, differences in the spectrum of phenazines produced may help define the ecological niche of the producing organism via effects on bacterial physiology as well as biological interactions with other microbes or hosts [2,3].

*P*. *chlororaphis (aureofaciens)* 30–84 was isolated for use in the management of take-all disease of wheat, and phenazine production by 30–84 is required for the inhibition of the causative agent, *Gaeumannomyces graminis* var. *tritici* [4]. *P*. *chlororaphis* 30–84 produces several phenazines, but only two in significant abundance: phenazine-1-carboxylic acid (PCA) and 2-hydroxy-PCA (2-OH-PCA) [4]. In liquid culture these may be produced at a ratio of 10:1, respectively [5]. In *P*. *chlororaphis* 30–84, as in most other phenazine-producing bacteria, the enzymes for the synthesis of the core phenazine PCA are encoded by a conserved set of biosynthetic genes *phzXYFABCD*, corresponding to the homologs named *phzABCDEFG* in *P*. *fluorescens* and *P*. *aeruginosa* [2,6]. Additionally, *phzO* located immediately downstream of the phenazine biosynthetic operon encodes a monooxygenase responsible for the hydroxylation of PCA to 2-OH-PCA [7]. Phenazine production responds to environmental conditions due to a complex regulatory network that includes two component systems (GacS/GacA and RpeA/RpeB), non-coding RNA (*rsmZ*), quorum sensing (PhzI/PhzR and CsaI/CsaR), sigma factor (RpoS) and other regulators (Pip, IopA/IopB) [8–12].

Phenazines also contribute to the ability of *P*. *chlororaphis* 30–84 to persist in the wheat rhizosphere [13]. Furthermore, phenazines produced by *P*. *chlororaphis* 30–84 are important for the formation of biofilm communities. For example, Maddula et al. [14] demonstrated using flow cell analysis that the *P*. *chlororaphis* 30–84 mutant 30-84ZN, which is deficient in phenazine production due to a *phzB*::*lacZ* insertion, was significantly impaired in its ability to form surface-attached biofilms compared to wild type. However, complementation of the phenazine defect via the introduction of the phenazine biosynthetic operon *in trans* resulted in extensive surface-attached biofilm formation. Furthermore, addition of purified phenazines to the growth medium restored biofilm formation by 30-84ZN, indicating the lack of phenazines were responsible for the deficiency in the capacity to form surface-attached biofilms. In subsequent experiments, Maddula et al. [5] generated derivatives of 30–84 that produced only PCA (30-84PCA), or overproduced 2-OH-PCA (30-84O*) via the deletion of the genomic copy of *phzO* or the over-expression of *phzO in trans*, respectively, (e.g. resulting in ratios of PCA to 2-OH-PCA in 30-84PCA, wild type and 30-84O* when grown in liquid culture of 10:0, 10:1, and 5:5, respectively). Single-pass flow cell assays showed that the 30–84 derivatives differed in their abilities to adhere to glass surfaces (30-84O* > 30–84 > 30-84PCA) and in the architecture of the surface attached biofilms they formed [5], demonstrating that the two phenazines differentially contribute to biofilm development and structure [15–17].

The biofilm matrix is composed of self-produced extracellular polymeric substances (EPS) that embed and surround bacterial cells, provide structural stability, and largely define the physiochemical environment within biofilms [17,18]. How the retention of different phenazines within the EPS influences gene expression patterns or biofilm matrix development remains to be determined. Recent studies showed that phenazines function in multiple ways in terms of their effect on biofilm development. For example, pyocyanin (PYO) produced by *P*. *aeruginosa* promotes extracellular DNA release by enhancing the generation of hydrogen peroxide in planktonic batch cultures [19]. The role of extracellular DNA (eDNA) as a structural component in biofilm architecture has been eloquently demonstrated [20]. Other studies showed that PYO binds to eDNA resulting in changes to bacterial cell surface properties [18], enhanced electron transfer capabilities [21], and increased viscosity of biofilm supernatants [21].

In the present study, we use a static culture assay (rather than flow cell or planktonic assays) to study the transcriptomic consequences of producing and retaining different phenazines within non-mixing cultures, focusing especially on genes that may be important for the generation of floating biofilm matrix, such as those potentially involved in eDNA release. Similar to our study, previous transcriptomic studies of bacterial “biofilms” have focused on non-attached biofilms formed during growth in static culture because a) of the difficulty in studying bacteria in surface attached biofilms and b) the finding that similar to surface attached biofilms, the global gene expression patterns of cells from static “biofilm” cultures are distinctly different from those grown in stationary phase planktonic cultures [22]. Additionally, the matrix-encased aggregates resemble surface attached biofilms in terms of their ability to protect cells from harsh environments and in the presence of eDNA in the EPS matrix [23]. These studies demonstrate the utility of using static biofilm-forming cultures to investigate the regulation of genes important for the establishment of the biofilm matrix and lifestyle.

In order to gain a more complete understanding of the impacts of phenazines on bacterial behavior and fitness, it is important to understand why strains produce more than one phenazine derivative and in particular how each of these contribute to the biofilm lifestyle. In the present study, the use of *isogenic derivatives* of *P*. *chlororaphis* 30–84 (e.g. wild type, 30-84O*, 30-84PCA, and 30-84ZN) provided the ability to examine *the relative contribution of different phenazines* to the biofilm phenotypes and gene expression patterns. We show that altering the phenazines produced by *P*. *chlororaphis* 30–84 differentially influences gene expression patterns, eDNA release, and biofilm matrix production.

## Materials and Methods

### Bacterial strains and growth conditions

Bacterial strains and primers are listed in Table 1. Liquid AB minimal medium supplemented with 2% casamino acids (AB + 2% CAA) (Difco, Becton Dickinson and Company, Franklin Lakes, NJ) was used for culturing *P*. *chlororaphis* as described previously [8].

**Table 1:** Bacterial strains and primers used in this study.

	Relevant characteristics^a^	Source
**Strains**		
***P*. *chlororaphis* 30–84**	Phz^+^Rif^R^ wild-type	[4]
**30-84PCA**	PCA^+^, 2-OH-PCA^-^, Rif^R^, Km^R^*phzO*::Tn5	[5]
30-84O*	2-OH-PCA^-^ overproducer	[5]
**30-84ZN**	Phz^-^Rif^R^*phzB*::*lacZ* genomic fusion	[10]
**Primers**		
**fliCRT1**	CTGCAAATCGCTACCCGTAT	[24]
**fliCRT2**	GAACAGCCAGTTCACGCATA	[24]
**katA1**	CCCACTTCAACCGTGAAAAC	This study
**katA2**	CGCAGGAAGGTAGGAGTCTG	This study
**ahpC1**	CCAGGGATTTCGTGATCAAT	This study
**ahpC2**	CAGACGGCTTCATCCTTGTC	This study
**phzO1**	TGGACCTCATACAGCCATTG	This study
**phzO2**	CCGCAAGCTGCACTATTTC	This study
**antA1**	AGCCCTTGTAGCTGTCGATG	This study
**antA2**	AACCAGTCCACCTTCACCTG	This study
**argC1**	ACCTGGACCTACGGTTTCG	This study
**argC2**	GTAGTCCTTCGGCAGCAGTC	This study
**lrgA1**	CCGAGATGCTGCTGTTCTTC	This study
**lrgA2**	ACACCCATTCCACCGTGA	This study
**phrS1**	CAGGAGGCCAGTCATGTTTT	This study
**phrS2**	GTGCTCGTTACCGGAAAGACT	This study
**hol1**	CATGCCCATCTGGCTTGT	This study
**hol2**	GTCGAGACCCCACAGATCAC	This study
**rpoDRT1**	ACGTCCTGAGCGGTTACATC	[8]
**rpoDRT2**	CTTTCGGCTTCTTCTTCGTC	[8]
**16SRT1**	ACGTCCTACGGGAGAAAGC	[8]
**16SRT2**	CGTGTCTCAGTTCCAGTGTGA	[8]
**US-F-EcoRI**	GGAATTCCTCGACGGTTCAGAGGGTTG	This study
**US-R-KpnI**	GGCGTCCGAGGTACCGTTTGTCATGTCACTCCTCC	This study
**DS-F_KpnI**	ATGACAAACGGTACCTCGGACGCCTGAACAACCGCC	This study
**DS-R-HindIII**	CCCAAGCTTGTCAGCGCCTTGACCGATG	This study
**KanR-F-KpnI**	CGCGCGCGGTACCTGTGTCTCAAAATC	This study
**KanR-R-KpnI**	CGCGCGCGGTACCTTTAGAAAAACTCATCG	This study
**Holin300-Up-F**	CTCGACGGTTCAGAGGGTTG	This study
**Holin-Dwn-R**	CCGGCTTTGTAGAGCTC	This study
**Holin-S-F**	GGAATTCCCAGGAGGAGTGACATGACAAAC	This study
**Holin-M-F**	GCGTATCGATCTGGACC	This study
**Holin-E-R**	CCCAAGCTTTTGTTCAGGCGTCCGAGCG	This study
**Holin500-F-EcoRI**	GGAATTCAGCCCATAACGCCAAAG	This study
**Holin500-R-HindIII**	CCCAAGCTTTTGTTCAGGCGTCCG	This study

^a^Km^R^ and Rif^R^ = kanamycin and rifampin resistance.

### Quantification of biofilm matrix and eDNA

For biofilm cultures, bacteria were grown in plates without shaking using an established method with modifications [22]. Pre-cultures were prepared overnight in shaking glass tubes (200 rpm) filled with 3 mL AB + 2% CAA at 28°C. The pre-cultures then were diluted to an OD_600_ of 0.05 in AB + 2% CAA and 1.5 ml per well were added to 12 wells of 24-well polystyrene Corning^®^ Costar^®^ cell culture plates (Corning Inc., Corning, NY, USA). The biofilm plates were sealed with an air-permeable cover and placed in an incubator at 28°C for up to 72 h. At 48 h, non-attached aggregates began to form. At different time intervals, the entire non-adhering 1.5 mL static cultures were transferred from the 24-well plates to Eppendorf tubes and cells collected by centrifugation (16,000 x g for 5 min). The supernatants were discarded and the mass of the cells and hydrated matrix were measured. The concentration of extracellular double stranded DNA was determined quantitatively using a Qubit 2.0 Fluorometer (Invitrogen Life Technologies, CA, USA). Briefly, static cultures were diluted 1:10 in water and vortexed to thoroughly disrupt the EPS. After centrifugation (16,000 x g for 5 min), the supernatants were transferred to new tubes and the remaining bacterial cells were removed via a 0.22 μm filter (Millipore, MA, USA). The concentration of eDNA was quantified by mixing 10 μl of supernatant with DNA quantifying fluorescent dyes from Qubit (Invitrogen Life Technologies). The fluorescence of DNA-dye interaction was measured using a Qubit 2.0 Fluorometer according to the manufacturer's instructions. The amount of eDNA was determined via comparison to a standard curve and reported as μg/ml.

For DNase treatment, 30 units of water-dissolved DNase I (Qiagen, Venlo, Netherlands) were added into bacterial cultures, and the mass of biofilm matrix and the amount of eDNA release were quantified after 60 h as described in this section. Equal volumes of water were used as a negative control.

### Phenazine extraction and quantification

Phenazine extraction was performed as described previously with modifications [9]. Briefly, triplicate 10-ml bacterial cultures of wild type and 30-84PCA were centrifuged (1,250 x g), and the supernatants were acidified with concentrated HCl. Separation of PCA and 2-OH-PCA were achieved by modifying the pH. Phenazines were extracted with an equal volume of benzene, mixed for 1 h in the rotary shaker, and centrifuged at 1,250 x g for 15 min. Nine milliliters of the benzene phase was transferred to new tubes, and evaporated under a stream of air. Phenazines were resuspended in 0.5 ml of 0.1N NaOH, and serial dilutions were quantified via absorbance at 367 nm (PCA) and 484 nm (2-OH-PCA). The amounts of PCA and 2-OH-PCA were determined by multiplying their absorption maxima by their standard extinction coefficients [5] and comparison to a standardized curve.

For exogenous phenazine treatment, each purified phenazine was added directly into the medium to the final concentration of 20 μg/ml. Overnight culture of 30-84ZN were prepared in shaking glass tubes (200 rpm) filled with 3 mL AB + 2% CAA at 28°C. The overnight cultures then were diluted to an OD_600_ of 0.05 in AB + 2% CAA amended with or without each phenazine and 1.5 ml per well were added to 12 wells of 24-well polystyrene Corning^®^ Costar^®^ cell culture plates. The mass of biofilm matrix and eDNA production were quantified as described above after 72 h of growth at 28°C without shaking.

### RNA preparation for transcriptomic analysis and qPCR

Two biological replicates of strains were started from single colonies grown on AB + 2% CAA and transferred to 1.5 ml AB + 2% CAA broth. All cultures were grown at 28°C in 24-well plates without shaking for 48 h to OD_600_ = 1.8 as described above. This time point coincided with the development of floating extracellular matrix in all of the derivatives tested. This method allowed us to observe the transcriptomic consequences of producing phenazines in a static culture and examine how each of the phenazines differentially contributes to matrix development.

RNA extractions were performed as described previously [24] with minor modifications. Briefly, 1 mL of the static cultures was collected and transferred to 15 ml polypropylene tubes (BD Bioscience, San Jose, CA) containing 2 ml RNA Protect reagent (Qiagen). Cells were harvested at this time point because biofilm matrix was actively being produced by all derivatives, although the amount of structured matrix formed varied among the derivatives. The cells harvested using this method included those in the static culture and those adhering to or embedded within the floating extracellular matrix. The 3 ml mixtures were vigorously vortexed for 1 min and then incubated at room temperature for 5 min to stabilize the RNA. Cells were harvested by centrifugation for 10 min at 4,000 × g and RNA was extracted using the Qiagen RNeasy Protect Bacteria Mini as recommended by the manufacturer. On-column DNA digestion was performed using Qiagen DNase. RNA was quantified using a Nano-Drop ND-100 spectrophotometer (NanoDrop Technologies; Wilmington, DE, U.S.A.) and RNA quality was checked using the Agilent 2100 Bioanalyzer (Agilent Technologies, Santa Clara, CA). Elimination of contaminating DNA was confirmed via qPCR amplification of the *rpoD* and *fliC* genes with SYBR green® dye on an ABI 9400HT PCR machine (Life Technologies, Carlsbad, CA). Ribosomal RNA (rRNA) was depleted from ~5 μg of total RNA using the RiboZero rRNA depletion kit (for Gram-negative bacteria, Epicentre Biotechnologies, Madison, WI). RNA quantification was achieved using a GE NanoVue Plus spectrophotometer (GE Healthcare Bio-Sciences Corp, Piscataway, NJ) and RNA quality was monitored with an Agilent 2100 Bioanalyzer (Agilent Technologies) at the Texas A&M Genomics and Technology Laboratory.

### Transcriptomic data analysis

RNA-Seq analysis was performed by Otogenetics, Atlanta, GA. Strand-specific cDNA libraries were constructed using Illumina TruSeq RNA Sample Preparation Kits. Based on gene annotation information, reads were mapped to the genome resulting in a compressed binary version of the Sequence Alignment Map (BAM files). To determine the transcriptional abundance for each gene, the number of reads that mapped within each annotated coding sequence (CDS) was determined. The number of reads per kb of transcript per million mapped reads (RPKM) was used to normalize the raw data and mean RPKM values were determined [25]. The complete dataset from this study has been deposited in the National Center for Biotechnology Information Gene Expression Omnibus with the Accession No.GSE61200. The mean RPKM values for each phenazine-producing derivative were standardized to the RPKM values of the phenazine non-producing derivative 30-84ZN to compute a ratio of mean RPKM values for each gene, and these were displayed by gene order from the chromosome origin of replication. Comparisons were performed using EdgeR [26] and genes with differences in gene expression based on the ratio were considered for further analysis when the *p*-value < 0.05 and the expression ratio was ≥ 2.0 or ≤ -2.0 (S1 Table).

### Quantitative PCR Methods and Analysis

Quantitative PCR was performed as described previously [27–29] RNA was reverse-transcribed using random primers (Invitrogen) and Superscript III (Invitrogen) at 50°C for 1 h and inactivated at 75°C for 15 min. SYBR Green reactions were performed using the ABI 7900 HT Fast System (Applied Biosystems, Foster City, CA) in 384 well optical reaction plates. Aliquots (1 μl) of cDNA (2 ng/reaction) were used as template for qPCR reactions with Fast SYBR Green PCR Master Mix (Applied Biosystems) and primers (500 nM final concentration). Primer pairs katA1-katA2, ahpC1-ahpC2, phzO1-phzO2, antA1-antA2, argC1-argC2, lrgA1-lrgA2, phrS1-phrS2, hol1-hol2, fliCRT1-fliCRT2, rpoDRT1-rpoDRT2 and 16SRT1-16SRT2 were used to detect the expression of *katA*, *ahpC*, *phzO*, *antA*, *argC*, *lrgA*, *phrS*, *hol*, *fliC*, *rpoD* and 16S rRNA genes, respectively (Table 1). The qPCR amplifications were carried out at 50°C for 2 min, 95°C for 10 min, followed by 40 cycles of 95°C for 15 sec and 60°C for 1 min, and a final dissociation curve analysis step from 65°C to 95°C. Three technical replicates of each of two biological replicates were used for each experiment. Amplification specificity for each reaction was confirmed by the dissociation curve analysis [25]. Ct values determined by the software were then used to calculate ΔΔCt for further analysis. The *rpoD* and 16S rDNA genes were used as the reference genes to normalize samples and log-transformed relative quantification (RQ) values were calculated for each gene with the control group as a reference [27–29].

### Construction of a Holin Mutant

A *P*. *chlororaphis* 30–84 holin mutant was generated using Flp recombinase, as described by Hoang et. al. [30]. The sequences 300 nt flanking the holin gene (pchl3084_1194) were amplified by two-step PCR using the primer pairs us-F-EcoRI, us-R-KpnI and ds-F-KpnI, ds-R-HindIII, respectively (see Table 1 for primers). Using the primer pair us-F-EcoRI and ds-R-HindIII with the contents of the previous PCR resulted in a product that contained the upstream fragment separated from the downstream fragment by a *Kpn*I restriction site. This fragment was ligated into pEX18Ap using the *Eco*RI and *Hin*dIII enzymes [30]. The kanamycin resistance cassette with its promoter was then PCR amplified using the primer set, KanR-F-KpnI and KanR-R-KpnI and pUC4K as the template. The product of the kanamycin cassette PCR was digested with *Kpn*I using commercial instructions and ligated between the upstream and downstream fragments in pEX18Ap. The final construct was electroporated into 30–84 and transformants were plated onto LB amended with Km. Double crossover mutants were obtained by counter-selection with LB amended with Km and 5% sucrose and confirmed using the PCR primers Holin300-Up-F, Holin-Dwn-R and the internal primers Holin-S-F, Holin-M, Holin-E-R. *In trans* complementation of this mutation was considered lethal because no cells expressing the construct were obtained after three attempts of cloning it into pGT2 [31] or pUCP20Gm [32] with the primers Holin500-F and Holin500-R, likely the result of multiple copy number.

### Transmission Electron Microscopy

Transmission electron microscopy (TEM) was performed at the Microscopy and Imaging Center at Texas A&M University. Specimens were observed on a JEOL 1200EX TEM operating at an acceleration voltage of 100 kV. Images were recorded at calibrated magnifications by CCD camera, and measurements were acquired using Image J software [33].

### Statistical analyses

Comparisons of matrix and eDNA production between derivatives were analyzed statistically using Analysis of Variance (ANOVA) and protected Least Significant Difference (LSD) tests (P < 0.05) (SAS Version 9.2, SAS Institute, Cary, NC).

## Results

### 2-OH-PCA enhances extracellular biofilm matrix and eDNA release

**Fig 1:**
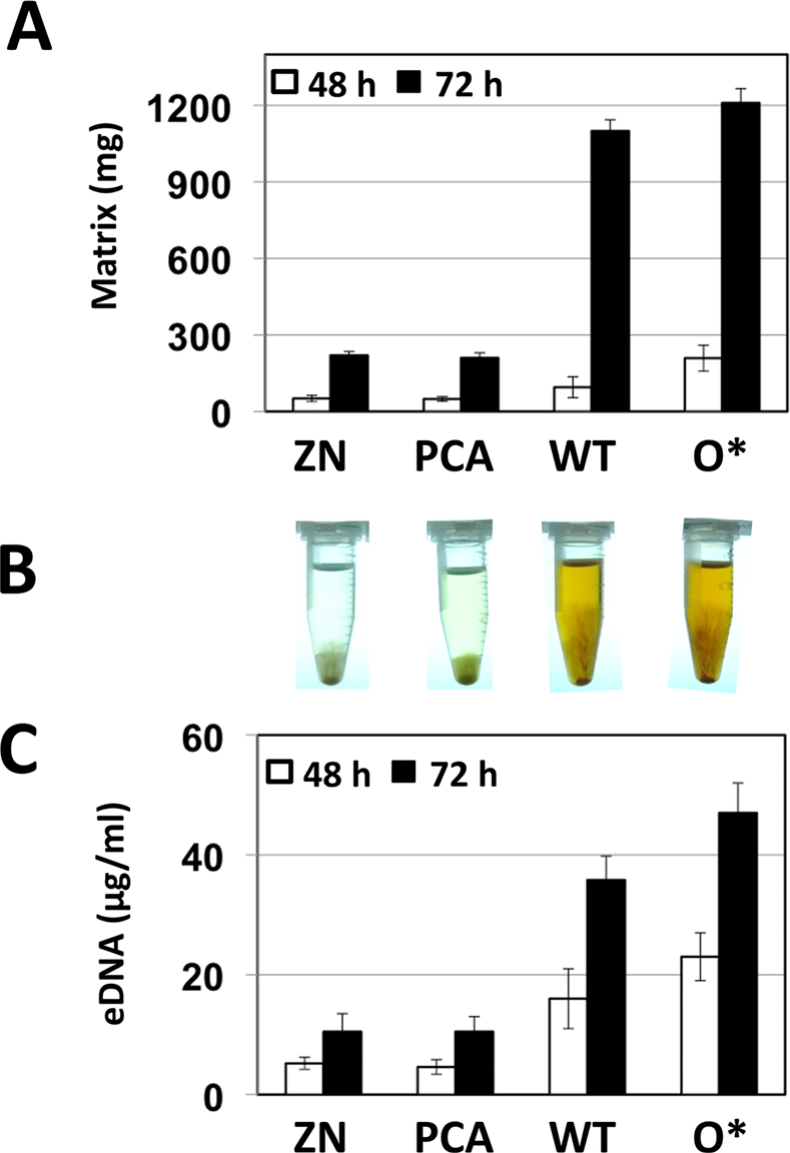
Production of non-attached biofilm and eDNA by 30-84ZN (no phenazine) 30-84PCA, 30–84 wild type, and 30-84O*. (**A)** Bacteria were grown in AB minimal media + 2% casamino acid in static plates for up to 72 h. Biofilm matrix production by *P*. *chlororaphis* 30–84 and derivatives at 48 and 72 h was quantified by weight. (**B)** Visualization of biofilm formed after culturing in static plates for 72 h and being collected by centrifugation. (**C)** Production of eDNA by different derivatives was quantified using the double stranded DNA quantifying fluorescent dye assay from Invitrogen. The data are the average of three biological replicates and error bars indicate the standard deviation.

Liquid cultures of *P*. *chlororaphis* 30–84 wild type, 30-84ZN, 30-84PCA and 30-84O* were grown in AB + 2% CAA in 24-well plates at 28°C for up to 72 h without shaking. At 48 h, the bacterial cultures of all 30–84 derivatives formed both surface attached and floating biofilms, the latter composed of extracellular matrix that varied in viscosity among the 30–84 derivatives. The entire, non-attached content of each well was transferred to an Eppendorf tube and the extracellular matrix was collected by centrifugation (S1 Fig). Electron microscopy revealed that bacterial cells were embedded within the extracellular matrix collected (S1 Fig), consistent with the definition of floating or non-adherent biofilms [15]. The structured, extracellular matrix that could be separated from the supernatant was quantified by weight (Fig 1A and 1B). The amount of biofilm matrix (mass) produced by 30-84ZN and 30-84PCA at both 48 and 72 h was similar. In contrast, biofilm matrix production by the two 2-OH-PCA producers, wild type and 30-84O*, was more than 5 times greater than 30-84ZN and 30-84PCA by 72 h. We hypothesized that eDNA may be a major component of the structured matrix.

To determine whether the production of PCA or 2-OH-PCA controlled the release of eDNA, quantitative fluorescent assays were performed comparing eDNA production by wild type, 30-84O*, and 30-84PCA to the phenazine defective mutant 30-84ZN (Fig 1C). The amount of eDNA produced by 30-84ZN and 30-84PCA at both 48 and 72 h was similar suggesting that endogenously-produced PCA alone does not enhance eDNA production. In contrast, eDNA production by the wild type was more than 3 times greater than the amount produced by 30-84ZN, especially at 72 h. This indicated that the production of 2-OH-PCA promoted eDNA production. Consistent with this hypothesis, the amount of eDNA produced by 30-84O* was 20% more than wild type.

To further confirm the role of phenazines in eDNA release and matrix production, 30-84ZN was monitored over 72 hours in the presence of exogenously-supplied PCA or 2-OH-PCA (20 μg/ml). Addition of 2-OH-PCA to the 30-84ZN cultures increased the amount of eDNA from 13.4 ± 3.9 μg/ml to 51.1 ± 8.8 μg/ml at 72 h post inoculation, whereas addition of PCA increased the amount to 39.15 ± 4.1 μg/ml. The mass of the biofilm matrix produced by 30-84ZN with 2-OH-PCA or PCA was greater than the amount produced by the 30-84ZN control (701.2 ± 56.8 mg, 423.7 ± 11.2 compared to 130.2 ± 28.0 mg, respectively). These results suggested that both exogenously-supplied PCA and 2-OH-PCA promote the production of eDNA and structured extracellular matrix.

### eDNA is a key structural component of the biofilm matrix

**Fig 2:**
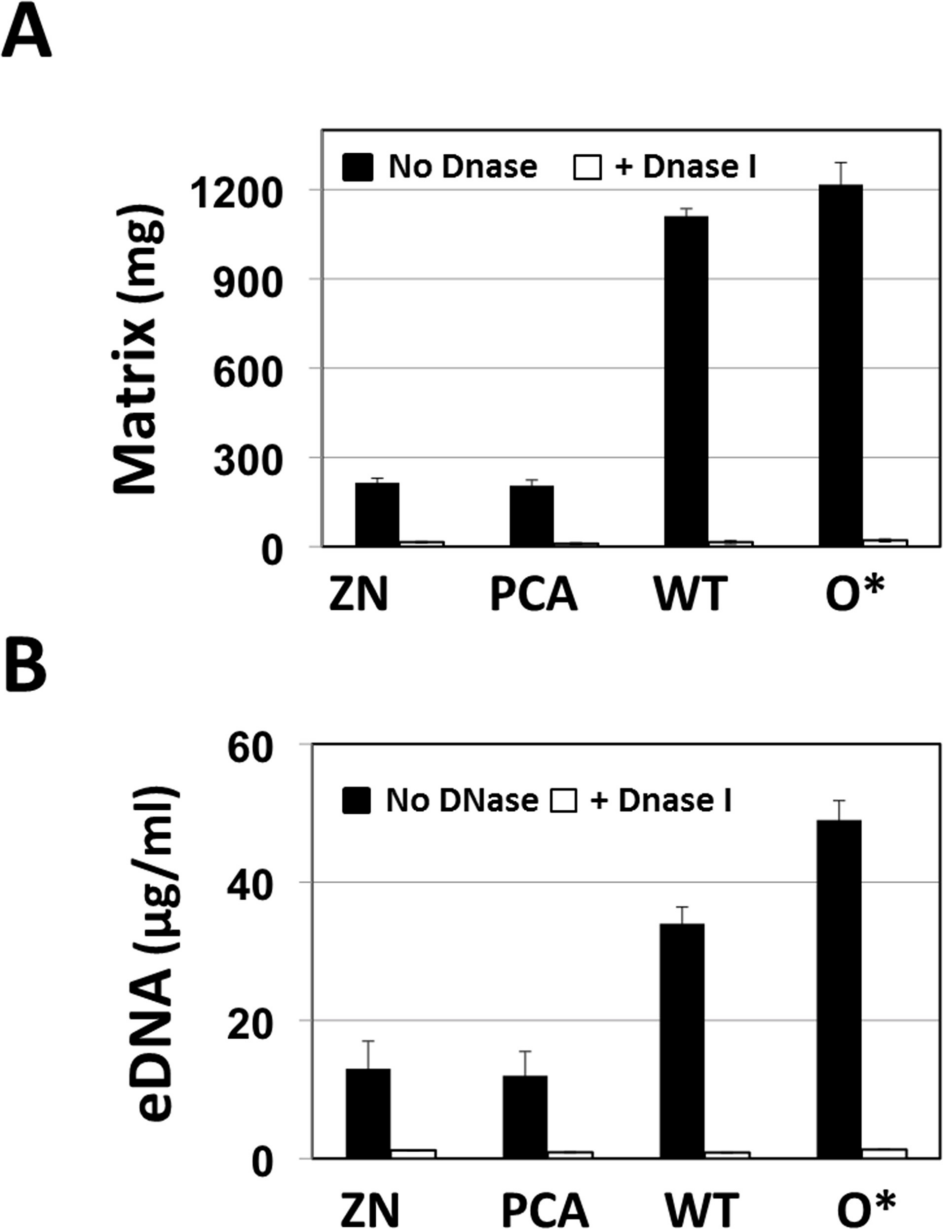
Production of biofilm matrix and eDNA in the presence or absence of DNase I. (**A)** Biofilm production after bacterial strains were grown in static plates in AB minimal media + 2% casamino acid either with or without DNase I for 72 h and biofilms were collected by centrifugation. Data are the weight of the biofilm matrix. (**B)** Production of eDNA in the presence or absence of DNase I in the same experiment. The data represent the average of at least three biological replicates and error bars indicate the standard deviation.

To determine whether eDNA plays a major role in maintaining the structure and hence our ability to collect and quantify the extracellular matrix, bacterial cultures of wild type, 30-84ZN, 30-84PCA and 30-84O* were incubated in the presence of DNase I. As shown in Fig 2A, growth with DNase reduced the quantity of extracellular mass that could be collected from cultures of all 30–84 derivatives. Removal of eDNA by DNase I was confirmed by quantitative fluorescent assays (Fig 2B). Together, these results demonstrate the essential roles of eDNA in the production/structure of the *P*. *chlororaphis* biofilm matrix.

### Transcriptome analysis and verification by qPCR

Transcriptomic analyses were performed to examine the regulatory effects of different phenazines on global gene expression patterns during biofilm matrix production. The transcriptome profiles of static culture populations of wild type, 30-84PCA and 30-84O* were compared to those of the control, e.g. the phenazine-defective mutant 30-84ZN as ratios of the mean RPKM values: those for which P value <0.05 and differ by more than two fold are shown in S1 Table. Henceforth a ratio ≥ 2 (Phenazine producer: 30-84ZN) indicates genes with enhanced or increased expression in the presence of phenazine; similarly a ratio ≤ -2 (30-84ZN: Phenazine producer, negative sign) indicates genes with reduced expression in the presence of phenazines.

**Fig 3:**
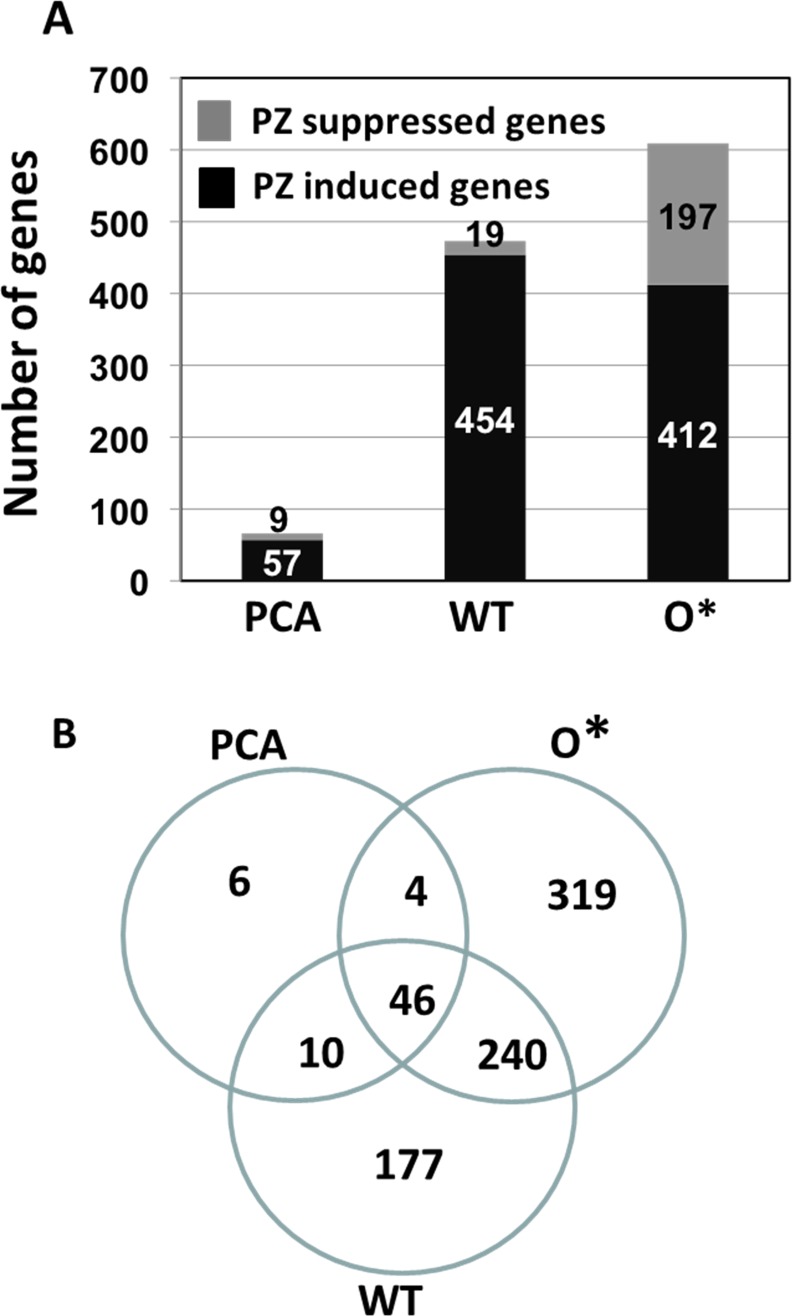
The number of differentially expressed genes in 30-84PCA, wild type and 30-84O* compared with the 30-84ZN. Differential expressed genes are those exhibiting over twofold change and a P value < 0.05. (**A)** Phenazine Induced and Suppressed genes are those that are expressed in at a higher or lower level, respectively, by the phenazine producing strains compared to 30-84ZN. (**B)** Venn diagram showing the number of genes differentially expressed in 30-84PCA, wild type and 30-84O* compared with 30-84ZN.

RNA-Seq analyses identified a total of 802 genes that were differentially expressed by the phenazine producing derivatives compared to 30-84ZN. PCA production caused minimal changes in the *P*. *chlororaphis* 30–84 transcriptome (Fig 3A). However, increasing the ratio of 2-OH-PCA had a broader impact on the bacterial transcriptome (Fig 3A). The transcript abundances of a total of 66 genes were differentially expressed by 30-84PCA compared to 30-84ZN, whereas a larger number of genes were differentially expressed by wild type and 30-84O* (473 and 609 genes, respectively, Fig 3A). Of these genes, a core set of 46 genes were differentially expressed by all phenazine producers (e.g. 30-84PCA, wild type and 30-84O*, Fig 3B). These results suggest that over-expression of 2-OH-PCA had far-reaching consequences on bacterial gene transcription in *P*. *chlororaphis* 30–84 (S1 Table).

Two-step qRT-PCR was performed on nine genes that were differentially expressed in the RNA-Seq analysis to validate the data. Four genes were commonly altered in 30-84PCA, wild type and 30-84O*: *katA* (catalase), *ahpC* (hydrogen peroxide reductase), *phzO* (phenazine biosynthesis) and *phrS* (regulatory RNA). Four genes were differentially expressed in wild type and 30-84O*, but not 30-84PCA: *antA* (anthranilate 1,2-dioxygenase), *argC* (N-acetyl-gamma-glutamyl-phosphate reductase), *lrgA* (regulator of cell lysis) and *hol* (holin). One gene was specifically altered in expression in 30-84O*, but not in 30-84PCA or wild type: *fliC* (flagellin). For all of the selected genes, the transcriptional fold changes measured by qRT-PCR were comparable to those obtained by RNA-Seq analyses (S2 Fig).

### Genes differentially expressed by all phenazine-producers

The core set of 46 genes that were differentially expressed in the matrix-forming cultures of 30-84PCA, wild type and 30-84O* (e.g. either higher or lower expression compared to 30-84ZN) included genes annotated as being involved in oxidative stress response, phenazine biosynthesis, drug resistance, and DNA repair (Table 2). We were particularly interested in the oxidative stress response genes because phenazines are well-known to induce oxidative damage in both prokaryotic and eukaryotic organisms [19,34]. Furthermore, phenazines are hypothesized to contribute to extracellular DNA release by enhancing the generation of hydrogen peroxide and thus the autolysis of cells [19]. To cope with the adverse effects of reactive oxygen species (ROS), bacteria have evolved multiple mechanisms including the production of ROS scavenging enzymes. In *Pseudomonas*, the most effective ROS detoxification enzymes include the major catalases KatA, KatB and KatG as well as the alkyl hydroperoxide reductases AhpC and AhpF [35]. Additionally, TrxB, a thioredoxin-disulfide reductase, protects protein disulfide bonds from oxidation [36]. Mutations in these genes greatly reduced ROS resistance and the ability to form biofilms in various *Pseudomonas* species [35,36]. Compared to the low levels of expression of these ROS-related genes in 30-84ZN (Table 2), phenazine production resulted in significantly higher expression of *ahpCF*, *katABG* and *trxB* genes. Interestingly, expression of the ROS-related genes was highest by wild type, perhaps indicating that production of both phenazines creates a more potent or diverse redox environment.

**Table 2:** Selected genes that are differentially expressed by all phenazine producers: e.g. by 30-84PCA, wild type, and 30-84O*compared with 30-84ZN.

Gene ID	Gene	Protein description	Ratio* (PCA/ZN)	Ratio* (WT/ZN)	Ratio* (O*/ZN)
**Oxidative stress**					
**Pchl3084_3280**	*ahpC*	alkyl hydroperoxide reductase	63.4	164.0	92.7
**Pchl3084_3279**	*ahpF*	alkyl hydroperoxide reductase	92.6	212.0	104.4
**Pchl3084_5293**	*katA*	catalase	8.3	18.3	10.3
**Pchl3084_5138**	*katB*	catalase	24.9	91.4	47.7
**Pchl3084_3902**	*katG*	catalase/peroxidase	6.6	15.1	8.8
**Pchl3084_0942**	*trxB*	thioredoxin-disulfide reductase	6.1	9.4	6.9
**Phenazine biosynthesis**					
**Pchl3084_4955**	*phzB*	phenazine biosynthesis protein	2.0	2.54	4.0
**Pchl3084_4956**	*phzC*	phenazine biosynthesis protein	289.2	351.2	605.3
**Pchl3084_4957**	*phzD*	phenazine biosynthesis protein	118.3	151.7	281.0
**Pchl3084_4958**	*phzO*	phenazine biosynthesis protein	42.6	119.6	216.3
**Efflux pump**					
**Pchl3084_3721**	*emrA*	multidrug resistance	2.9	7.4	5.8
**Pchl3084_3720**	*emrB*	multidrug resistance	2.8	5.6	5.3
**Other genes**					
**Pchl3084_1311**	*phrS*	PhrS RNA	-5.8	-6.8	-3.5

*Ratios ≥ 2 (Mean RPKM Phenazine producer/ mean RPKM 30-84ZN) indicate genes with increased expression in the presence of phenazines. Ratios ≤ -2 (30-84ZN/ Phenazine producer, negative sign) indicate genes with reduced expression in the presence of phenazines.

Other genes expressed at higher levels in the phenazine-producing strains compared to 30-84ZN included the phenazine biosynthetic genes (*phzBCD* and *phzO*), which likely is due to disruption of phenazine biosynthesis by the *phzB*::*lacZ* mutation in 30-84ZN (Table 2). Additionally, two genes, *emrA* and *emrB*, annotated as encoding a multidrug resistance efflux system were notably expressed (2.5–7 fold increase) in the phenazine-producing derivatives relative to 30-84ZN (Table 2). Whether this system is functional or plays a role in antimicrobial transport or intrinsic resistance to phenazines remains to be determined. In contrast, the expression of the small RNA PhrS was notably reduced by phenazine production (3–6 fold). In *P*. *aeruginosa*, PhrS activates the expression of *pqsR*, which in turn stimulates synthesis of the quinolone signal [37]. In *P*. *chlororaphis* 30–84, the potential targets of PhrS remain unknown since both PqsR and the quinolone signaling pathway are absent. The significance of these gene expression changes is currently under investigation.

### Genes differentially expressed by 2-OH-PCA-producing derivatives

A total of 240 genes was changed in expression (relative to 30-84ZN) only in wild type and 30-84O* biofilm matrices, e.g. may be affected specifically by 2-OH-PCA production (Fig 3B). The expression of the majority of these genes (222) was enhanced by 2-OH-PCA production. One group of positively regulated genes is annotated as bacteriophage-derived, R2-type pyocin genes (Pchl3084_1194 to Pchl3084_1233). This 13 Kb gene cluster harbors genes encoding pyocin structural proteins involved in R type pyocin assembly as well as a holin and lytic protein involved in cell lysis for pyocin release. The expression of the gene cluster was increased by 2-OH-PCA production (Table 3). These results suggest that the influence of 2-OH-PCA on eDNA production is due in part to cell autolysis as a result of pyocin production and release.

**Table 3:** Genes annotated as R2 type pyocin genes belonging to a 13 Kb open reading frame spanning a bacteriophage-derived gene cluster from Pchl3084_1194–1233.

Gene ID	Gene	Protein description	Ratio*	Ratio*
			(WT/ZN)	(O*/ZN)
**R2 type pyocin genes**				
**Pchl3084_1194**	*Hol*	pyocin R2, holin	3.1	8.7
**Pchl3084_1195**		N-acetylmuramoyl-L-alanine amidase domain protein	2.8	5.5
**Pchl3084_1196**	*llpA*	putidacin L1	3.0	5.7
**Pchl3084_1197**		hypothetical protein	2.8	5.4
**Pchl3084_1198**		phage baseplate assembly protein V	2.8	5.5
**Pchl3084_1199**		hypothetical protein	2.8	5.6
**Pchl3084_1200**		baseplate J family protein	2.5	5.3
**Pchl3084_1201**		phage tail protein I	2.9	5.7
**Pchl3084_1202**		phage tail collar domain protein	2.7	5.4
**Pchl3084_1203**		putative phage tail protein	2.9	5.2
**Pchl3084_1204**		hypothetical protein	3.0	4.9
**Pchl3084_1205**		putative phage tail sheath protein	2.9	4.7
**Pchl3084_1206**		putative phage tail tube protein	2.9	4.7
**Pchl3084_1207**		hypothetical protein	3.2	5.2
**Pchl3084_1208**		hypothetical protein	3.1	5.7
**Pchl3084_1209**		phage tail tape measure protein, TP901 family	2.9	5.7
**Pchl3084_1210**		phage protein, P2 GpU family	2.8	5.6
**Pchl3084_1211**		putative tail protein X	2.8	5.5
**Pchl3084_1212**		phage protein, late control D family	2.9	5.5
**Pchl3084_1213**		hypothetical protein	3.0	5.9
**Pchl3084_1214**		hypothetical protein	3.2	6.4
**Pchl3084_1215**		putative tail sheath protein	3.2	5.8
**Pchl3084_1216**		hypothetical protein	3.4	5.3
**Pchl3084_1217**		hypothetical protein	3.3	5.4
**Pchl3084_1218**		putative phage tail protein	3.6	5.8
**Pchl3084_1219**		DNA circularization, N-terminal domain protein	3.2	6.0
**Pchl3084_1220**		putative bacteriophage Mu P protein	3.2	6.3
**Pchl3084_1221**		bacteriophage Mu Gp45 protein	3.6	6.7
**Pchl3084_1222**		phage protein, GP46 family	3.5	6.4
**Pchl3084_1223**		baseplate J family protein	3.2	6.1
**Pchl3084_1224**		hypothetical protein	3.2	6.0
**Pchl3084_1225**		hypothetical protein	3.3	6.7
**Pchl3084_1226**		hypothetical protein	3.1	6.1
**Pchl3084_1227**		putative phage tail protein	3.2	6.8
**Pchl3084_1228**		putative phage tail protein	3.3	7.0
**Pchl3084_1229**		putative phage tail protein	3.3	7.0
**Pchl3084_1230**		putative phage tail protein	3.2	7.1
**Pchl3084_1231**		putative pyocin R, lytic enzyme	3.4	7.2
**Pchl3084_1232**		hypothetical protein	3.4	7.5
**Pchl3084_1233**	*cinA*	competence/damage-inducible protein CinA	1.6 *ns*	2.6
**Cell lysis regulation**				
**Pchl3084_0954**	*lrgA*	regulator of cell lysis	-2.4	-2.3
**Pchl3084_0955**	*lrgB*	regulator of cell lysis	-2.3	-2.5

These genes are either highly expressed in 30-84O* or 30-84O* and wild type, but not 30-84PCA compared with 30-84ZN. Also included are the cell lysis regulator genes *lrgAB* located upstream of the cluster.

*Ratios ≥ 2 (mean RPKM Phenazine producer/ mean RPKM 30-84ZN) indicate genes with increased expression in the presence of phenazines. Ratios ≤ -2 (Mean RPKM 30-84ZN/ Mean RPKM Phenazine producer, negative sign) indicate genes with reduced expression in the presence of phenazines. *ns* indicates this value is not significant.

**Fig 4:**
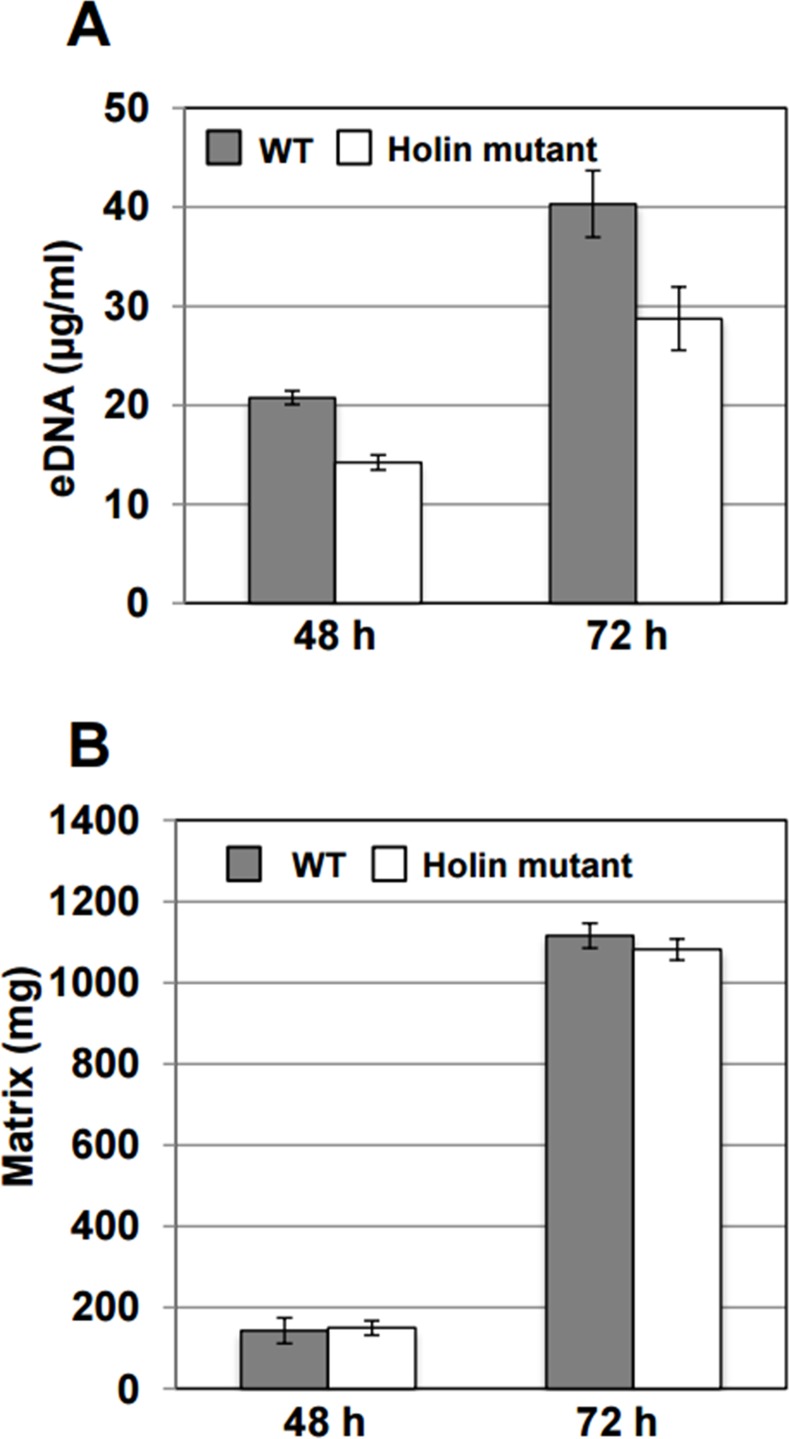
The effect of holin mutation on eDNA and biofilm matrix production. (**A)** Production of eDNA by *P*. *chlororaphis* 30–84 wild type and the holin mutant after growing 48 and 72 hr in static culture. (**B)** Biofilm matrix production by 30–84 wild type and the holin mutant at 72 h. The data are the average of three biological replicates and error bars indicate the standard deviation.

To clarify the role of the pyocin gene cluster in eDNA release, the holin-encoding gene of the pyocin gene cluster was replaced with a Km^R^ cassette. Holins are small transmembrane proteins that facilitate cell lysis during bacteriophage infection. Holins accumulate and permeabilize the cytoplasmic membrane, which allows the passage of a muralytic enzyme into the periplasmic space, which degrades the peptidoglycan layer resulting in osmotic lysis [38]. We hypothesized that if the holin in the pyocin gene cluster was a major contributor to eDNA production, its deletion would inhibit cell lysis because the muralytic enzyme would not be able to reach and degrade the peptidoglycan layer. Indeed, the 30–84 holin mutant released less eDNA into the biofilm matrix compared to wild type after 48 and 72 hours (Fig 4A). However, eDNA release was not eliminated by the deletion of the holin encoded in the pyocin gene cluster, indicating that 30–84 possesses other mechanisms for eDNA release (see Discussion). Interestingly, the decrease in eDNA was not associated with a significant decrease in matrix mass (Fig 4B). This may be because the amount of eDNA produced, although reduced, is still sufficient for matrix formation.

RNA-Seq also revealed other genes activated by 2-OH-PCA that are involved in the production of secondary metabolites or exoenzymes. The transcript abundances of four genes *prnA*, *prnB*, *prnC* and *prnD* annotated as pyrrolnitrin biosynthesis genes were significantly (~3 fold greater in biofilm matrices of the 2-OH-PCA producing derivatives, wild type and 30-84O*, compared to 30-84ZN, whereas these genes were not differentially expressed in 30-84PCA biofilm matrices (Table 4). The expression of *aprA* (extracellular protease), *lsc* (levansucrase), and chitinases encoding genes were higher (~2–18 fold) in wild type and 30-84O* compared to 30-84ZN biofilm matrices (Table 4).

**Table 4:** Selected genes that were changed in expression (relative to 30-84ZN) only in wild type and 30-84O* biofilm matrices, e.g. are specifically affected by 2-OH-PCA production.

Gene ID	Gene	Protein description	Ratios* (WT/ZN)	Ratios* (O*/ZN)
**Exoenzymes and Secondary metabolites**				
**Pchl3084_3146**	*prnA*	tryptophan halogenase	2.8	3.4
**Pchl3084_3145**	*prnB*	pyrrolnitrin biosynthesis	2.6	3.0
**Pchl3084_3144**	*prnC*	halogenase	2.6	3.0
**Pchl3084_3143**	*prnD*	aminopyrrolnitrin oxidase	2.6	3.6
**Pchl3084_2021**	*chiC*	chitinase	2.6	5.5
**Pchl3084_3180**	*chiC*	chitinase	6.1	17.6
**Pchl3084_3127**	*aprA*	metalloprotease	2.6	2.6
**Pchl3084_4797**	*lsc*	levansucrase	3.9	12.0
**Efflux pumps**				
**Pchl3084_3193**	*mexE*	multidrug efflux RND transporter, membrane fusion protein	5.4	5.6
**Pchl3084_3194**	*mexF*	multidrug efflux RND transporter, permease protein	5.2	4.6
**Pchl3084_3195**	*oprN*	multidrug efflux RND transporter, outer membrane factor	2.6	3.1
**Pchl3084_3327**	*oprM*	outer membrane efflux protein	2.3	2.4
**Pchl3084_3329**	*mexA*	multidrug resistance protein	3.6	2.4
**DNA repair**				
**Pchl3084_0814**	*recN*	DNA repair protein	2.3	3.8
**Pchl3084_1932**	*lexA*	*lexA* repressor	2.0	2.7
**Pchl3084_2827**	*ligD*	DNA ligase D	2.2	2.4
**Pchl3084_3586**	*dnaE2*	DNA polymerase	2.2	5.2
**Pchl3084_3589**	*lexA2*	*lexA* repressor	2.6	5.1
**Pchl3084_3851**		DNA endonuclease	2.4	2.2
**Pchl3084_5136**	*radA*	DNA repair protein	4.5	2.6
**Iron uptake**				
**Pchl3084_0982**	*fecR*	sigma factor regulatory protein FecR	3.5	4.0
**Pchl3084_0983**	*fecI*	RNA polymerase sigma factor FecI	3.7	4.7
**Pchl3084_3208**	*efeU*	ferrous iron permease	2.7	4.2
**Pchl3084_4064**	*pvdS*	sigma-70 factor	4.5	6.3
**Metabolism**				
**Pchl3084_0747**	*antC*	dioxygenase reductase	-3.8	-5.3
**Pchl3084_0748**	*antB*	dioxygenase	-3.9	-7.2
**Pchl3084_0749**	*antA*	dioxygenase	-4.0	-6.1
**Pchl3084_3869**	*catA*	catechol 1,2-dioxygenase	-2.5	-4.1
**Pchl3084_3870**	*catC*	delta-isomerase	-2.1	-2.7
**Pchl3084_3879**	*argC*	glutamyl-phosphate reductase	-4.7	-11.4
**Pchl3084_2275**	*argD*	acetylornithine transaminase	-4.8	-2.5
**Pchl3084_4622**	*argO*	arginine exporter protein	2.4	2.4
**Pchl3084_0036**	*trpB*	tryptophan synthase	-2.2	-4.1
**Pchl3084_1486**	*iaaH*	indoleacetamide hydrolase	-2.1	-6.1

*Ratios ≥ 2 (mean RPKM Phenazine producer/ mean RPKM 30-84ZN) indicate genes with increased expression in the presence of phenazines. Ratios ≤ -2 (Mean RPKM 30-84ZN/ Mean RPKM Phenazine producer, negative sign) indicate genes with reduced expression in the presence of phenazines.

Transcripts for genes encoding efflux pumps, DNA modification genes, and certain genes involved in iron uptake also were more abundant in the biofilm matrices of the 2-OH-PCA producing derivatives. In addition to EmrAB, two other efflux pumps were increased in expression specifically in the presence of 2-OH-PCA production. The transcript abundances of the *mexA-oprM* and *mexEF-oprN* genes were ~2–5 fold greater in wild type and 30-84O* as compared to 30-84ZN (Table 4). These efflux pumps transport different antimicrobial compounds in other pseudomonads. For example, the MexEF-OprN RND efflux pump transports fluoroquinolones, trimethoprim, and chloramphenicol [39], whereas the MexAB-OprM system transports quinolones and β-lactams [40]. Transcript abundance of other ROS/DNA repair genes also were increased (2–5 fold) in the presence of 2-OH-PCA, including *recN* (DNA repair protein), *lexA* (*lexA* repressor), *ligD* (DNA ligase), *dnaE2* (DNA polymerase), *radA* (DNA repair protein), and Pchl3084_3851 (nuclease) (Table 4). Finally, the transcript abundances of *fecR* and *fecI* genes were enhanced (3–4 fold) in the presence of 2-OH-PCA as well as several FecR-controlled genes such as *pvdS*, *efeU* and *TonB* family genes. FecR and FecI are known for their role in regulating iron uptake genes [41].

**Fig 5:**
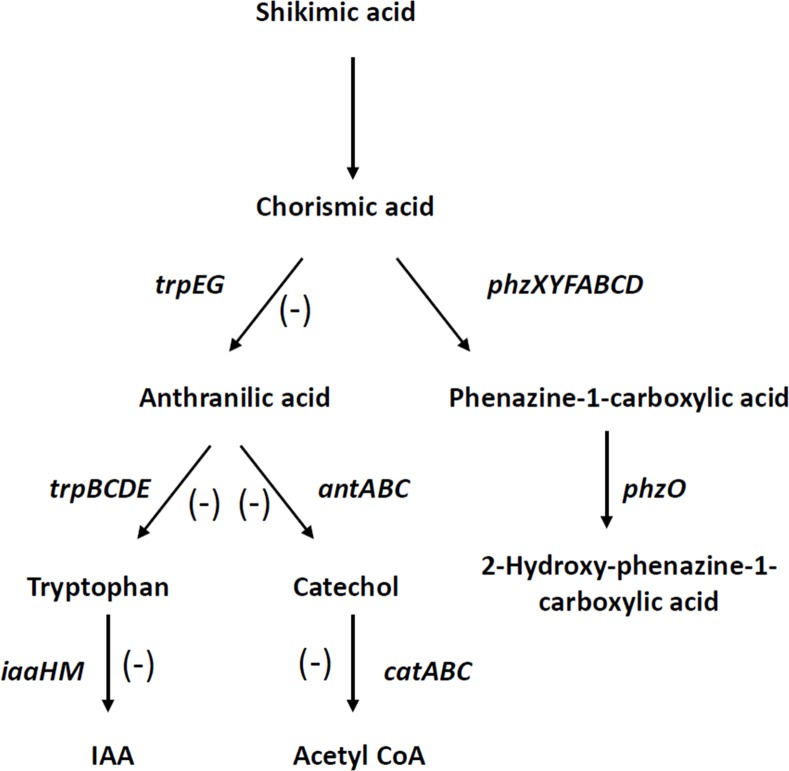
Possible metabolic fates for chorismic acid in *P*. *chlororaphis* 30–84. Chorismic acid is produced via the shikimic acid pathway and is a metabolic precursor for a number of different end products in pseudomonads, including phenazines (indicated by solid lines). The negative symbols (-) indicate competing pathways in which transcript levels for key genes are reduced in 2-OH-PCA producing strains compared to 30-84ZN (Table 4).

The genes in the 2-OH-PCA producing derivatives wild type and 30-84O* with reduced transcript abundance (relative to 30-84ZN) are annotated as being involved in anthranilate metabolism (Table 4). These include the *antABC* operon (conversion of anthranilate to catechol), *catAC* (conversion of catechol to acetyl-CoA), *trpB* (conversion of anthranilate to tryptophan) and *iaaH* (conversion of tryptophan to indole acetic acid). Phenazine and anthranilate biosynthesis compete for the metabolic pool of chorismic acid and many of the genes reduced in expression in the 2-OH-PCA producers are involved in the biosynthesis of acetyl-CoA and IAA utilizing anthranilate as a common substrate (Fig 5).

## Discussion

Many phenazine-producing strains produce more than one phenazine. *P*. *chlororaphis* 30–84 produces predominantly PCA and 2-OH-PCA. These phenazines are essential for the biological control capabilities of 30–84, including pathogen inhibition, rhizosphere competence, and biofilm formation. However these capabilities are influenced significantly by the ratio of the two primary phenazines produced, suggesting the two phenazines differentially contribute to these competencies [5]. In this study, we examined how different phenazine ratios affected eDNA and biofilm matrix production by comparing the phenotypes of wild type and derivatives 30-84O*, and 30-84PCA when grown in static culture to those of the control 30-84ZN. We also examined the transcriptomic consequences of producing different phenazines at the 48 h time point (e.g. when all derivatives were producing matrix). Although broad impacts of phenazine production on gene expression patterns were revealed, we limit discussion to genes that are potentially relevant to matrix production and lifestyle in the floating biofilm.

The production of 2-OH-PCA by wild type and 30-84O* stimulated eDNA release and extracellular matrix production compared to the phenotypes observed for 30-84PCA and 30-84ZN. Moreover, addition of 2-OH-PCA or PCA to the growth medium of 30-84ZN stimulated eDNA release and extracellular matrix production by this phenazine non-producing derivative, with eDNA production and matrix formation being greatest in the presence of 20 μg/ml of 2-OH-PCA compared to the presence of the same amount of PCA. Significantly, we found that eDNA was an important structural component of the floating matrix as evidenced by the drastic reduction in the mass of matrix that could be collected following treatment with DNase I.

Multiple lines of data support the hypothesis that the eDNA detected in the 30–84 biofilms originated in part from the autolysis of a subpopulation of biofilm cells. One cause of autolysis may be the generation of Reactive Oxygen Species (ROS) as phenazines accumulated in the static cultures. It is well documented that phenazines are responsible for the generation of ROS, which contribute to host virulence and the competitive inhibition of other microorganisms, including plant pathogenic fungi [34]. Recently, the phenazine pyocanin produced by *P*. *aeruginosa* was shown to contribute to eDNA release by enhancing the generation of hydrogen peroxide, and subsequently cell lysis [19]. Consistent with phenazine-induced generation of hydrogen peroxide and ROS, transcriptomic analysis showed that expression levels of several ROS detoxifying enzymes as well as a number of DNA repair/modification enzymes were significantly higher in the phenazine producing strains compared to the control 30-84ZN. These results suggest that both PCA and 2-OH-PCA production cause oxidative stress in 30–84 biofilm matrices. Expression of several of these genes was slightly enhanced in 2-OH-PCA strains coincident with the enhanced eDNA production, suggesting that 2-OH-PCA may be more potent than PCA as a determinant of ROS and hence eDNA production.

Transcriptomic analysis revealed that additional genes related to cell autolysis were highly induced specifically by 2-OH-PCA, suggesting a linkage between phenazine production and specific cell autolysis mechanisms. Autolysis of cells in biofilm development was reported previously in *P*. *aeruginosa* [42], where a pathway to cell autolysis is through the action of holins and their associated muralytic enzymes [43]. Holins are phage-encoded small integral membrane proteins that control the activity of murein hydrolases and the thus the timing of host cell lysis during bacteriophage replication. Holins initiate the formation of pores in the cytoplasmic membrane enabling cytoplasmic murein hydrolases to reach and degrade the peptidoglycan layer, causing cell lysis and release of bacteriophage or pyocin [38,44]. In the 2- OH-PCA producing derivatives, autolysis due to the increased expression of the holin-containing R2 pyocin gene cluster is likely to have contributed to the large amount of eDNA observed in these biofilms. However, results indicate this is not the only source of eDNA; other sources may be related to the presence of additional holins or other unidentified mechanisms. Analysis of the 30–84 genome revealed that it also harbors homologs of *cidA* and *lrg*, the holin and antiholin genes found in *P*. *aeruginosa* PAO1. The holin CidA is hypothesized to be involved in ROS mediated cell death [38], whereas the antiholin LrgA neutralizes the activity of CidA in PAO1 biofilms [17,45]. Although expression of the *cidA* homolog was unchanged by 2-OH-PCA production, expression of *lrg* antiholin homolog was reduced two-fold by 2-OH-PCA production (Table 3). The potential role of these homologs in eDNA production is currently under investigation. Consistent with 2-OH-PCA inducing the expression of the pyocin gene cluster, preliminary results demonstrated that a bacteriocin capable of killing an indicator *Pseudomonas* species is induced under matrix-forming conditions (data unpublished). Whether the bacteriocins observed in the biofilm killing assays are the R2 type pyocin identified via transcriptomic analysis as induced by 2-OH-PCA is currently under investigation. The results of this study provide evidence for a linkage between 2-OH-PCA production and the induction of pyocin gene expression and eDNA release.

The transcriptomic analysis also revealed that the expression of genes encoding pathways that compete with intermediates needed for phenazine biosynthesis were lower in phenazine-producing derivatives, especially 2-OH-PCA-producing derivatives, relative to 30-84ZN. For example, phenazine production was coincident with the reduced expression of genes in several pathways involved in anthranilate metabolism, including genes involved in acetyl-CoA and IAA biosynthesis (Fig 5). It was shown previously that the *P*. *chlororaphis* phenazine biosynthetic gene *phzF* encodes a homolog of DAHP synthases that enhances the availability of the key metabolic intermediate chorismic acid for phenazine biosynthesis by bypassing tryptophan inhibition of the shikimic acid pathway [46]. Given the organization of the *P*. *chlororaphis* phenazine operon (e.g. *phzXYFABCD*), 30-84ZN with its disruption of *phzB* has a functional *phzF*, but does not produce phenazines. Lower expression of chorismate-competing anthranilate metabolism pathways in phenazine-producing strains compared to 30-84ZN is consistent with the hypothesis that phenazine production channels metabolic intermediates away from these pathways. Of note 30-84ZN mutants produce more indole acetic acid than wild type (unpublished).

Moreover, RNA-Seq analysis revealed that phenazine production, and in particular 2-OH-PCA production, was correlated with higher transcript levels of genes involved in the production of some exoenzymes and secondary metabolites known to be produced by 30–84 [47]. For example, the expression of *aprA* (extracellular protease), *lsc* (levansucrase), and two genes encoding chitinases were higher in wild type and 30-84O* static cultures compared to 30-84ZN. Additionally, the expression of *prn* genes annotated as involved in pyrrolnitrin biosynthesis was enhanced in 30-84O*. However, pyrrolnitrin production remains to be demonstrated in 30–84, perhaps because planktonic cultures rather than static or biofilm populations were screened for pyrrolnitrin production. The higher transcript levels associated with phenazine production are consistent with the hypothesis that phenazines act as signals for the coordinated production of selected secondary metabolites and exoenzymes that may contribute to ecological fitness within the biofilm niche, perhaps via activation of ROS mediated signaling pathways. It is conceivable that the formation of biofilms limits the dispersion of many secondary metabolites once they are outside the cell, thereby concentrating their effectiveness while reducing their cost in terms of loss. Thus, their coordinate regulation with phenazine production may be timed to coincide with the production of a structured biofilm matrix during the switch to a sessile lifestyle.

It is now well understood that phenazines serve multiple roles in the ecological fitness of the producing microbe and that these roles may vary according to the type (s) of phenazines produced and the environments the phenazine-producing microbes inhabit. Recent work with *P*. *aeruginosa* indicates a previously unrecognized role for the phenazine pyocyanin in promoting eDNA release, which likely plays an important role in shaping the biofilm environment both structurally and functionally with relevance to infection. In the present study, we show that phenazine production by *P*. *chlororaphis* 30–84, and 2-OH-PCA production in particular, induces the release of eDNA and matrix production, suggesting that phenazine induction of eDNA release and the contribution of both phenazine and eDNA to structured matrix production may be common among phenazine-producers. In *P*. *chlororaphis* 30–84, phenazine production is requisite for competitive persistence in the rhizosphere of plants, which likely is contingent on the conditioning of the local environment via biofilm matrix production for survival. What is less well understood is why many phenazine producers including *P*. *chlororaphis* 30–84 and *P*. *aeruginosa*, produce more than one type of phenazine and in particular why the different derivatives are produced in specific proportions. This study demonstrates that the production of *different phenazines* by derivatives *of the same organism* differentially affects the production of floating biofilm matrix as well as the associated gene expression patterns. Currently, it is unclear whether the ratio of phenazines produced by *P*. *chlororaphis* 30–84 naturally varies in response to the presence of other phenazine producers or environmental factors and how such stimuli affect the biofilm-related genes identified here. Studies to evaluate biofilm production under these conditions are currently underway. The results of the present study expand the current understanding of the functions different phenazines play in the behavior and fitness of bacteria in biofilm-forming communities. Importantly, our results reveal broad phenotypic and transcriptomic consequences related to the production of 2-OH-PCA.

## Supporting Information

S1 Fig Biofilm formation by *P*. *chlororaphis* 30–84 wild type grown in static culture. **A**. Demonstration of floating biofilm matrix produced by *P*. *chlororaphis* 30–84 wild type. Bacteria were grown without shaking in 24 well plates for 72 h. After brief centrifugation, the bacterial aggregate was visualized using a 1 ml tip. **B**. Transmission electron microscopy of the aggregate. Cells were negatively stained with 1% phosphotungstic acid (pH 7.0) and micrographs were taken at an accelerating voltage of 100 kV.
(TIF)

S2 Fig Verification of RNA-Seq data by quantitative reverse transcription polymerase chain reaction (qRT-PCR). The relative fold change (log_2_) of each gene was derived from the comparison of 30-84PCA, wild type and 30-84O* to the 30-84ZN phenazine-deficient mutant. The 16S rDNA gene was used as an endogenous control. The relative fold changes are reported as the means of three replicates. The experiments were repeated twice with similar results. Error bars indicate standard deviation.
(TIF)

S1 Table Genes that are differentially expressed by at least one of the phenazine producers: e.g. by wild type, 30-84O*, or 30-84PCA compared to 30-84ZN. The gene locus tag, gene name (if known), gene product (if known) as provided by the most recent GenBank annotation are given. The mean RPKM values (ZN AVERAGE, WT AVERAGE, O* AVERAGE, PCA AVERAGE) for each strain (30-84ZN, 30–84 wild type, 30-84O*, and 30-84PCA, respectively). Ratios ≥ 2 (Mean RPKM Phenazine producer/ mean RPKM 30-84ZN) indicate genes with increased expression in the presence of phenazines. Ratios ≤ -2 (30-84ZN/ Phenazine producer, negative sign) indicate genes with reduced expression in the presence of phenazines. P values (PValue WT:ZN, PValue O*:ZN, PValue PCA:ZN) for each statistical comparison are provided. Genes are displayed in order from the chromosome origin of replication. Comparisons were performed using EdgeR [26] and genes with differences in expression were considered for further analysis when the *p*-value < 0.05 and the expression ratio was ≥ 2.0 or ≤ -2.0).
(XLSX)
