# Supplementary figures and images for: The Phenazine 2-Hydroxy-Phenazine-1-Carboxylic Acid Promotes Extracellular DNA Release and Has Broad Transcriptomic Consequences in Pseudomonas chlororaphis 30–84
Source: PLoS One. 2016 Jan 26;11(1):e0148003. doi: 10.1371/journal.pone.0148003 (PMC4727817; doi:10.1371/journal.pone.0148003)

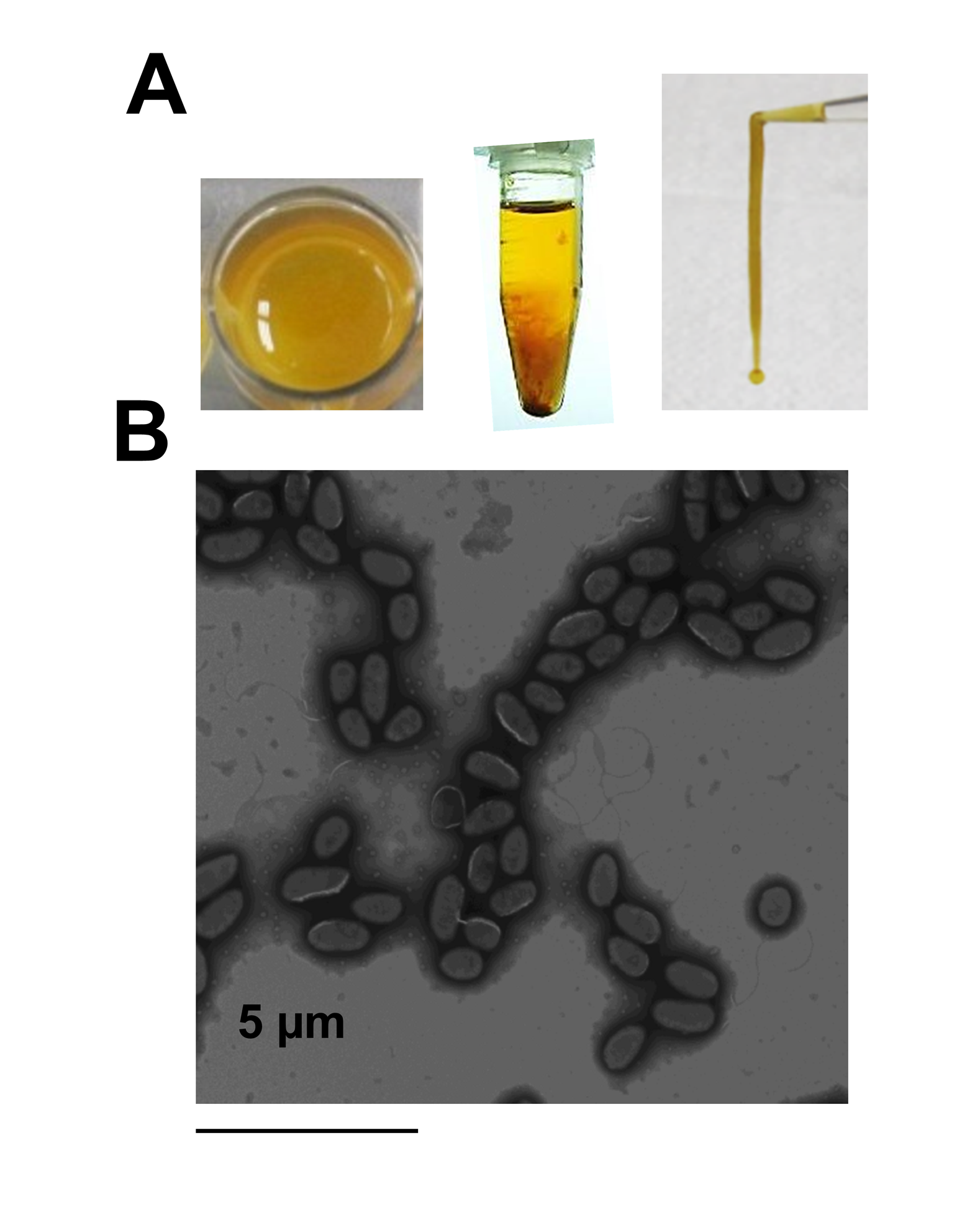

Supplement: S1 Fig — A. Demonstration of floating biofilm matrix produced by P. chlororaphis 30–84 wild type. Bacteria were grown without shaking in 24 well plates for 72 h. After brief centrifugation, the bacterial aggregate was visualized using a 1 ml tip. B. Transmission electron microscopy of the aggregate. Cells were negatively stained with 1% phosphotungstic acid (pH 7.0) and micrographs were taken at an accelerating voltage of 100 kV. (TIF) [file pone.0148003.s001.tif]

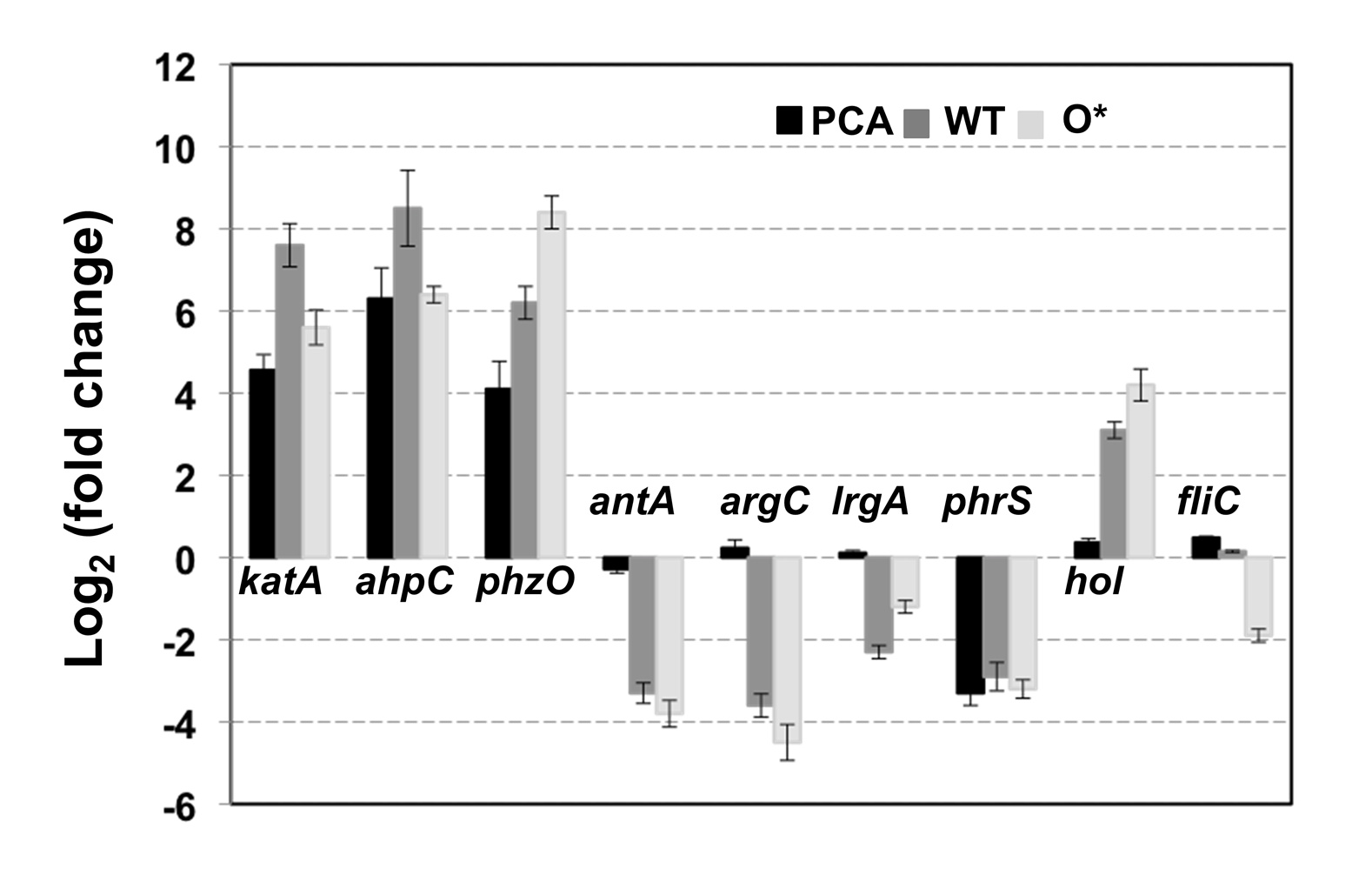

Supplement: S2 Fig — The relative fold change (log2) of each gene was derived from the comparison of 30-84PCA, wild type and 30-84O* to the 30-84ZN phenazine-deficient mutant. The 16S rDNA gene was used as an endogenous control. The relative fold changes are reported as the means of three replicates. The experiments were repeated twice with similar results. Error bars indicate standard deviation. (TIF) [file pone.0148003.s002.tif]
